# Academic Achievement in University Students: The Role of Perfectionism and Academic Hardiness

**DOI:** 10.5964/ejop.12755

**Published:** 2024-11-29

**Authors:** Iwanna Sepiadou

**Affiliations:** 1 Department of Psychology, Aristotle University of Thessaloniki, Thessaloniki, Greece; Dublin City University, Dublin, Ireland

**Keywords:** adaptive perfectionism, maladaptive perfectionism, academic hardiness, academic achievement, research article

## Abstract

The aim of this study was to investigate the relationship between two personality factors, namely perfectionism and academic hardiness, and academic achievement. Nine hundred sixty-six undergraduate students from diverse disciplines in Greece made up the entire sample. In addition to two self-reported questionnaires about their achievements, perfectionism, and academic toughness, they were asked to complete one demographic questionnaire. The study revealed statistically significant positive correlations between the adaptive form of perfectionism and academic achievement and negative primarily correlation between the maladaptive form of perfectionism and academic achievement. The dimensions of academic hardiness (challenge, commitment, control) were also found to be positively correlated with the students’ performance. Regarding the predictive role of these two factors for academic achievement, the results indicated that the adaptive form of perfectionism (high standards) and two dimensions of academic hardiness (challenge and commitment) are positive predictors, while the maladaptive form of perfectionism (discrepancy) is a negative predictor. Implications of the above results are discussed.

The present study attempts to provide empirical evidence for the factors that may influence academic achievement. According to the existing literature, the predictors of academic achievement seem to be complex. Academic achievement is considered one of the most important targets of education in the current cultural and socio-economic context (Bansal & Pahwa, 2015; Madigan, 2019). Thus, it constitutes a crucial point of the educational research. Uncovering the components that may affect academic achievement would help psychologists and educators to design and implement effective interventions for enhancing students’ performance.

Academic achievement refers to what a student has accomplished during the passage of the academic years regarding different subjects of studies (Ahmadi et al., 2013; Bansal & Pahwa, 2015). Academic achievement can be measured in various ways, such as accomplishing a specific goal within school or university, individual test performance, and performance across classes (grade point average, GPA). GPA is the most widely used indicator of academic success and is determined by averaging a student’s grades from all their classes over the academic year (Endleman et al., 2022), which is also used in the present study.

Review of the literature in this field suggested that there are several factors which have an impact on students’ academic performance. Some of the most important antecedents in this context are the factors relevant to the educational environment and circumstances, the factors relevant to the teachers or professors, and the factors that are related to the students (Ahmadi et al., 2013). Therefore, a wide range of variables have the potential to influence achievement. Knowledge of the impact of different factors on academic achievement is necessary for the teachers to maximize the students’ performance in a worthwhile manner (Bansal & Pahwa, 2015). Investigators in educational psychology suggest that personality factors may be crucial. Personality traits can improve the likelihood of aptitudes, attitudes, and behaviors that are contributing to better or worse achievement. Although considerable evidence has confirmed that personality traits, such as Conscientiousness and the other traits of the five-factor model, predict academic achievement (see Poropat, 2009; Richardson et al., 2012; Vedel, 2014), other personality variables have attracted limited research. The present study will provide evidence for the predictive value of two personality factors for academic achievement in higher education, namely perfectionism and academic hardiness, as the relevant research results so far remain largely inconsistent and/or scant.

## Perfectionism

Perfectionism is one personality trait that has been linked to academic success (Park et al., 2020; Stoeber, 2012; Stoeber et al., 2018). Perfectionism is the drive to strive for excellence and have high standards for oneself when working on a task; yet it is frequently associated with negative self-criticism (see Hill, 2016; Xie et al., 2018). Perfectionism has an impact on many facets of life. Being flawless is now viewed by society as a key sign of success, particularly in academic contexts. As a result, a lot of young people tend to think that to succeed well academically, they must be flawless (Curran & Hill, 2018; Stoeber, 2017).

Perfectionism is a multifaceted notion, thus the relationship between it and academic achievement appears to be complicated. Factor analytic studies have provided support for two higher-order dimensions, namely adaptive and maladaptive. Adaptive perfectionists are people who have high expectations, work hard to meet them, and can still feel content when they are fulfilled. High standards, little room for error, and a persistent fear of making mistakes and disappointing people are characteristics of maladaptive perfectionists (Rice et al., 2014; Stoeber, 2017; Stoeber, 2018; Stoeber & Otto, 2006). Many motivational, cognitive, emotional, and behavioral outcomes have been linked to perfectionism (see Stoeber, 2018). This is why it is critical to distinguish between perfectionistic dimensions since they demonstrate different and at times opposite patterns of relationships with several outcomes.

Academic achievement has been found to be connected with and/or predicted by perfectionism as a personality characteristic, based on relevant research data (see Akar et al., 2018; Hill, 2016; Park et al., 2020; Stoeber, 2012). However, the relationship between the two variables seems to vary based on the dimension of perfectionism examined. For instance, in a study contacted on 350 university students (see Akar et al., 2018), both dimensions of perfectionism were found to have an impact on students’ performance. Academic achievement was found to be positively predicted by adaptive perfectionism and negatively predicted by maladaptive perfectionism. Perfectionism is characterized by high standards, which are embodied in perfectionistic aspirations. These incredibly high expectations have to do with the motivating elements that govern and guide actions that lead to improved performance. Those who are adaptive perfectionists are more inclined to participate, endure, and dedicate more time to pertinent tasks. Thus, among the two main characteristics of perfectionism, adaptive perfectionism is more likely to be positively correlated with academic achievement, particularly when the relationship with the perfectionistic worries is controlled. Contrariwise, maladaptive perfectionism comprises critical evaluations and constant concerns about making mistakes. People with high levels of maladaptive perfectionism may worry more about making mistakes than about picking up new skills. Furthermore, they can put off engaging in pertinent educational activities in favor of putting off doing them in order to avoid failing. Hence, maladaptive perfectionism is more likely to negatively affect academic achievement (Hewitt & Flett, 1991; Stoeber, 2018; Stoeber et al., 2018). The picture, although, seems to be far more complex, since Park et al. (2020) found out that the two dimensions of perfectionism did not directly affect academic achievement, but only their indirect effects through accurate self-assessment were statistically significant. Thus, the present study aims to contribute to the existing literature by further investigating the aforementioned direct relationship and to promote a practical and balanced understanding of perfectionism in academic settings.

## Academic Hardiness

Benishek and Lopez (2001) used the phrase “academic hardiness” to investigate the reasons behind the inclination of certain students to embrace academic difficulties, such as challenging courses, while others choose to steer clear of them. Hardiness is conceptualized as a combination of three constructs, namely commitment, control, and challenge. The concept of commitment refers to someone’s belief that it is important to remain involved with the events and actively participate in certain activities or tasks, no matter how stressful the things are. The idea of control pertains to an individual’s perception of their ability to efficiently manage their time, exercise control over their education, and prioritize tasks that they deem significant. In conclusion, the idea of challenge pertains to the extent to which a person views demanding circumstances and challenging assignments as a chance for continued education rather than as a danger (Benishek et al., 2005; Benishek & Lopez, 2001; Cheng et al., 2019; Kobasa, 1979; Maddi, 2006).

It has been shown that academic hardiness and its components correlate with academic achievement (see Ahmadi et al., 2013; Kamtsios & Karagiannopoulou, 2013a, 2015; Sheard, 2009). More specifically, Kamtsios and Karagiannopoulou (2013b) conducted research on 478 Greek undergraduate students and found that there is a positive correlation between academic hardiness and students’ performance. In their study the concept of commitment was most highly positively correlated with academic achievement. Their findings are in agreement with those of Ahmadi et al. (2013) and Sheard (2009). Performance of hardy individuals is enhanced by their active or decisive coping efforts in stressful and difficult situations. Strong hardy attitudes in students are desirable, as hardiness facilitates turning difficulties to advantage, growing in such enhanced performance criteria as creativity and maintaining physical and mental health (Maddi, 2006). Nevertheless, there is currently little proof that intellectual toughness is a reliable indicator of academic success in university. To our knowledge, only one study has found that commitment constitutes a positive predictor of academic achievement (see Sheard, 2009). To date, there is no evidence to show the predictive role of control and challenge on academic achievement. This is somewhat surprising, given the overwhelming evidence of the beneficial effects of hardiness across a variety of occupations. The current research attempts to fill this gap and contribute to the existing literature regarding the relationship between academic hardiness and university students’ performance.

## The Present Study

This study is an extension of a previous study on the same population (see Sepiadou & Metallidou, 2023). The previous analysis examined the phenomenon of academic procrastination in university students and the factors that could predict it, while the present research examines whether the same factors could predict academic achievement, contributing to the existing literature and filling research gaps.

More specifically, taking into consideration the importance of academic achievement in the present socio-economic and cultural context, the current study aimed at providing not only empirical evidence for the correlation between two personality factors (perfectionism and academic hardiness) and academic achievement, but also for the possible predictive role of these factors for the students’ academic performance. Numerous studies have investigated the relationships personality factors show with academic achievement, the existing data regarding the impact of components of perfectionism and academic hardiness are limited or non-existent though. The present study could contribute to the existing literature by filling the gaps, and it would help us to better understand why certain students select hard assignments, engage in strategies to get through challenging coursework, control their emotions when they fail to meet their objectives, and generally have higher GPAs.

According to the existing literature (e.g., Akar et al., 2018; Hill, 2016; Park et al., 2020; Stoeber, 2012), it is expected that the adaptive dimensions of perfectionism (high standards and order) are positively correlated with the students’ academic achievement (**Hypothesis 1a**), while the maladaptive dimension of perfectionism (self-criticism/evaluative concerns) is negatively correlated with academic achievement (**Hypothesis 1b**). In addition, it is expected that the three dimensions of academic hardiness (commitment, control, and challenge) are positively associated with the students’ performance (**Hypothesis 2**). As regards the predictive role of perfectionism for academic achievement, adaptive dimensions of perfectionism are expected to be significant positive predictors (**Hypothesis 3a**), while the maladaptive dimension to be a significant negative predictor (**Hypothesis 3b**). Finally, based on research conducted by Sheard (2009), the dimension of academic hardiness, namely commitment, is expected to positively predict academic achievement (**Hypothesis 4**). Last but not least, we need to find out if the other two aspects of academic toughness—control and challenge—significantly predict students' success because there is a dearth of pertinent research data.

## Method

### Participants

The study included a sample of 966 undergraduate students, 242 of whom were men and 724 of whom were women. First-year students were excluded from this study since they had not yet completed a variety of academic activities and had an unclear grade point average (2nd year: *n* = 161, 3rd year: *n* = 198, 4th year: *n* = 234, and > 5th year: *n* = 373). The participants attended a variety of Greek higher education institutions, with the majority coming from the theoretical and technological departments of the National and Kapodistrian University of Athens (*N* = 205) and the Aristotle University of Thessaloniki (*N* = 315). The participants' average age was 23 years old (*M* = 23.07, *SD* = 4.7).

### Procedure

The questionnaires were distributed to different groups of university students via Google Forms and social media. Two online self-reported questionnaires and a demographic questionnaire were given to the participants to complete. They were all made aware of the study's objectives, the sample's characteristics, and its confidentiality protocols. There were no rewards for participating; it was entirely voluntary. They were also told that they could stop participating in the survey whenever they wanted. Lastly, they were told how to get in touch with them regarding any queries they might have or the findings once the study was completed.

The research complied fully with the American Psychological Association's code of ethics and the European Union Regulation on Sensitive Personal Data (GDPR; https://gdpr.eu/tag/gdpr/), which went into effect on May 25, 2018, and which is applicable in Greece under Law 4624/2019 (Issue A´ 137/29.08.2019). Every participant in the data coding process was assigned a digit as a name between 1 and 966.

### Materials

#### Demographics

Participants were asked to respond to several demographic questions (gender, age, year of study, higher education institution) prior to filling out the basic questionnaires.

#### Measure of Academic Achievement

Academic achievement was assessed with one question embedded in the demographics questionnaire regarding the students’ grade point average (GPA), which is considered the most common measure of academic performance.

#### Measure of Perfectionism

An adaptation of The Almost Perfect Scale Revised (Slaney et al., 2001) into Greek by Diamantopoulou and Platsidou (2014) was used to measure perfectionism. The twenty-three items on this self-reported questionnaire examine perfectionism as a multidimensional construct using three subscales. The first sub-scale (questions, such as “I have high expectations for myself”) asks about the respondent’s high standards of excellence and aspirations. One of the four items on the second sub-scale, “I like to always be organized and disciplined,” gauges the person's need for order. The third sub-scale counts the difference, or “gap,” that could exist between the individual’s initial standards of perfection and the outcome (12 items, such as “My best just never seems good enough for me”). Respondents rate themselves on a 5-point Likert scale, with the options “strongly disagree” to “strongly agree.” The tool’s three factors and acceptable psychometric qualities were well-supported by factor analysis in the current sample. A disparity with an eigenvalue of 7.41 explained 32.23% of the variation. Order, with an eigenvalue of 1.88, accounted for 8.17% of the variation, while high standards, with an eigenvalue of 4.33, explained 18.82% of the variance. For discrepancy the alpha coefficient was α = .94, for order α = .83, and for high standards α = .82.

#### Measure of Academic Hardiness

Academic hardiness was measured in the current study using a translated version of The Revised Academic Hardiness Scale, which was created by Benishek et al. (2005) and translated into Greek by Kamtsios & Karagiannopoulou (2013b). The forty items on the self-reported questionnaire evaluate students’ behavioral, cognitive, affective, and motivational views in relation to general and difficult academic situations. These elements approximately correspond to three categories: commitment, challenge, and control. The 13 items in the commitment scale, such as “I will not go out with my friends if I have to study for an exam,” gauge an individual’s propensity to stick with the tasks at hand, regardless of how demanding and stressful they may be. Challenge assesses a person’s propensity to view challenging assignments as a chance for more learning rather than as a danger (11 items, such as “I take difficult classes because I know they will benefit me in the future”). Control pertains to the capacity of the learner to manage feelings that may surface during assignments and impact the process of learning (16 items, for example, “I am good at calming myself down when I feel anxious about my ability to do well on a test or a class project”). Respondents rate items on a 4-point Likert scale, with “strongly disagree” and “strongly agree” being the possibilities. The current sample’s factor analysis produced the same three factors. A challenge whose eigenvalue was 8.65 explained 21.62% of the variation. With an eigenvalue of 5.21, commitment accounted for 13.02% of the variation, whereas an eigenvalue of 4.62 explained 11.56% of the variance for control. The alpha coefficients for commitment (α = .86), challenge (α = .82), and control (α = .87) were obtained.

## Results

### Preliminary Analyses, Descriptive Statistics, and Intercorrelations

First, complex variables were constructed, based on the factors highlighted by the factor analyses. Means, standard deviations, skewness, and kurtosis values are presented in Table 1. Measures of skewness and kurtosis were applied to verify whether parametric analyses could be applied on the data (see Sepiadou, 2023). Since all of the skewness and kurtosis values were less than 2, they were all regarded as normally distributed (see Kline, 2011).

**Table 1:** Means, Standard Deviations, Skewness, and Kurtosis of the Variables

Variable	*M*	*SD*	Skewness	Kurtosis
**Academic achievement**				
GPA	7.28	0.81	0.19	-0.03
**Perfectionism**				
Discrepancy	3.05	0.98	0.11	-0.86
Standards	3.86	0.73	-0.41	-0.33
Order	3.78	0.89	-0.54	-0.25
**Academic hardiness**				
Challenge	2.33	0.59	0.19	-0.61
Commitment	2.62	0.53	-0.21	-0.55
Control	2.49	0.64	-0.04	-0.66

A series of correlation analyses using the Pearson correlation coefficient was initially performed to investigate the bilateral relationships between the variables under consideration. The relationships between academic accomplishment, perfectionist traits, and academic toughness traits are shown in Table 2. The association between perfectionism's dimensions and academic achievement varies depending on which dimension is studied. To be more precise, academic success is inversely connected with the maladaptive form of perfectionism (discrepancy) and positively correlated with the adaptive form (high standards and order). In other words, their GPA rises in proportion to the degree to which they describe having high standards and enjoying order in their academic lives. Contrariwise, the more they report having a discrepancy between their goals and their accomplishments, the lower is their GPA. Furthermore, a positive correlation was observed between academic accomplishment and each of the three aspects of academic hardiness, with the characteristic of commitment having the strongest correlation. Their GPA thus increases with the degree to which students report being academically resilient. Additionally, Table 2 demonstrates that in the majority of instances, there are statistically significant relationships between various perfectionism aspects and academic toughness. Order positively connected with commitment and control, high standards positively correlated with challenge and commitment, and discrepancy negatively correlated with the commitment and control elements of academic hardiness. Only high standards were shown to be connected with the challenge dimension, suggesting a strong relationship between high personal standards and finding academic work to be interesting, demanding, and nonthreatening.

**Table 2:** Correlations Between Academic Achievement, Dimensions of Perfectionism, and Dimensions of Academic Hardiness

Variable	1	2	3	4	5	6	7
1.Academic achievement	–						
2. Discrepancy	-.119**	–					
3. Standards	.312**	.164**	–				
4. Order	.151**	-.105**	.369**	–			
5. Challenge	.311**	-.035	.277**	.034	–		
6. Commitment	.377**	-.205**	.490**	.430**	.284**	–	
7. Control	.125**	-.624**	-.012	.087**	.137**	.201**	–

**p < .01

### Regression Analyses

Using the Enter technique, linear regression analyses were carried out to investigate the potential predictive value of perfectionism and academic hardiness characteristics for academic attainment. First, the relationship between academic accomplishment and the three perfectionism dimensions—discrepancy, high standards, and order—is investigated. Discrepancy and high standards, two aspects of perfectionism, were found to be significant predictors of students’ achievement, *F*(3,961) = 46.931, *p* < .001, *R*^2^ = .128. To be more precise, it was discovered that high standards dimension (adaptive form of perfectionism) was a positive predictor (β = 0.338, *p* < .001), whereas discrepancy (maladaptive form of perfectionism) was, as predicted, a negative predictor of academic achievement (β = -0.175, *p* < .001). Regarding the predictive value of the dimensions of academic hardiness for academic achievement, two of the three dimensions were found to be significant predictors, *F*(3,961) = 74.458, *p* < .001, *R*^2^ = .189. Specifically, challenge and commitment were both positive predictors with commitment being a stronger predictor (β = 0.220, *p* < .001 and β = .308, *p* < .001 respectively). The control dimension was not a significant predictor (β = 0.034, *p* = .255).

## Discussion

This study's primary goal was to learn more about university students’ academic success by analyzing the impact of two personality traits, perfectionism and academic hardiness. Initially, it was examined the possible correlational relationship between the dimensions of perfectionism and students’ achievement. According to our findings, the adaptive form of perfectionism (high standards and order) is positively correlated with the students’ performance assessed by GPA, confirming our Hypothesis 1a. On the other hand, as it was expected, the maladaptive form of perfectionism (discrepancy) is negatively correlated with academic achievement (confirmation of Hypothesis 1b). High standards and order enclose perfectionistic strivings. These dimensions of perfectionism relate to motivational factors that will regulate behaviors which are conducive to better performance. In addition, high standards may mean that more time is spent on academic tasks, providing extra means for higher achievement (Stoeber et al., 2018). Contrariwise, discrepancy is a defining feature of perfectionism that is encapsulated by perfectionistic concerns. Students with high levels of perfectionistic concerns may spend less time in academic activities to avoid mistakes, failure, and criticism. Thus, it is maladaptive perfectionism that is likely to be negatively associated with students’ performance (Hewitt & Flett, 1991; Stoeber, 2018).

In the next step, we investigated the possible correlation between the dimensions of academic hardiness and academic achievement. All dimensions of academic hardiness (challenge, commitment, control) were positively associated with students’ achievement with the concept of commitment being most highly positively correlated with GPA. These findings are in total agreement with those of Kamtsios and Karagiannopoulou (2013b), confirming our second hypothesis. From a theoretical perspective, these results are expected, as hardy individuals tend to turn difficulties to advantage and cope with stressful situations. Therefore, hardy attitudes grow students’ creativity and enhance their performance (Maddi, 2006).

Moreover, in an attempt to understand the factors that may affect academic achievement, we investigated whether the dimensions of perfectionism and academic hardiness are predictors of students’ achievement. Based on the findings of previous research (see Akar et al., 2018; Hill, 2016; Stoeber, 2012), we postulated that the adaptive form of perfectionism—high standards and order—is a positive predictor of academic achievement (Hypothesis 3a), whereas its maladaptive form—discrepancy—is a negative predictor (Hypothesis 3b). Our Hypothesis 3a was partially confirmed, as only the dimension of high standards was found to predict positively the students’ achievement. Setting high standards means that students may be more engaged and more likely to persevere in dealing with difficult tasks, having consequently better performance (Stoeber et al., 2018). The results of the present study did not indicate a direct effect of order dimension on academic achievement. This finding is in accordance with that of Park et al. (2020), who found that perfectionistic strivings did not directly predict academic achievement, but only through inaccurate self-assessment. In addition, the findings of the current research confirmed our Hypothesis 3b. It appears that the maladaptive form of perfectionism may account for the lower students’ GPA. This finding is not surprising given that discrepancy between goals and accomplishments encloses perfectionistic concerns, which are likely to be detrimental for university students (Madigan, 2019).

Further, the results of the present study revealed that two dimensions of academic hardiness, namely challenge and commitment, constitute positive predictors of academic achievement, confirming the fourth Hypothesis (i.e., commitment dimension predicts positively students’ achievement). This finding is in agreement with that of Sheard (2009), whose study seems to be the only one which has indicated that one dimension of academic hardiness, commitment, predicts positively the students’ academic performance. Academic hardy students consider difficult tasks as an opportunity for further learning and not as a threat. It is important for them to participate and remain involved in academic activities and tasks, no matter how stressful they are (Benishek & Lopez, 2001; Benishek et al., 2005; Cheng et al., 2019; Kobasa, 1979; Maddi, 2006). Τhis attitude seems to lead to better academic performance.

### Limitations and Further Directions

Despite the aforementioned promising findings, only self-report questionnaires were used in the present study for the measurement of the variables. Although self-reports have the advantage of giving personal information inaccessible otherwise, they pose a limitation due to their subjectivity. Moreover, the study’s sample is mainly composed of women and previous researchers have found that female students have better academic performance than male students (see Fortin et al., 2015; Marcenaro–Gutierrez et al., 2018). While our sample did not reveal statistically significant differences between genders in academic accomplishment, the limited number of male students in comparison to female students may restrict the generalizability of the findings across genders. Furthermore, the only “student-related” personality characteristics of academic achievement are examined in this quantitative study. As a matter of fact, several elements, including those pertinent to professors and the educational environment and conditions, influence students’ academic success. All the aforementioned elements should be included in future research, along with more general contextual elements pertaining to the degree of education (undergraduate or graduate), the study program’s profile, and the area of competence. Qualitative studies using in-depth interviews with university students could uncover part of the interaction among the aforementioned factors. More longitudinal studies could be conducted to capture any potential dynamic interactions between situational and personal factors from the time students enter the program until they graduate. Finally, future studies could look into the combined consequences of the two perfectionism qualities. The proposed 2 x 2 model of perfectionism is one method that enables researchers to do this (see Gaudreau & Thompson, 2010). This concept adds to the discussion about whether perfectionism is maladaptive or adaptive. There is proof that this model is useful when considering academic accomplishment (Kljajic et al., 2017).

### Conclusion and Implications

Given the above limitations, the results of the present study contribute to a deeper insight into the possible factors that influence academic achievement. This paper suggests that two personality factors, perfectionism and academic hardiness, constitute helpful constructs to seize the individual differences in academic performance. It is our expectation that this study will help to fill the existing gap regarding the relationship between the dimensions of academic hardiness and academic achievement. In addition, the current research will contribute to the existing literature where the relevant data so far are inconsistent.

The findings of this study could be considered as a resourceful guideline for professional counselors and teachers or professors. We consider that future intervention programs for the enhancement of students’ academic performance should take into consideration the dimensions of perfectionism and academic hardiness. For instance, boosting participants’ academic hardiness could lead them to a better performance by being highly committed to difficult tasks and seeing them as an opportunity for further learning and not as a threat. Moreover, professional counselors and teachers should contribute and help students to set high but at the same time realistic standards. These exceptionally high standards relate to motivational factors that will target and control behaviors that are beneficial for a better performance. Students with high standards are considered as adaptive perfectionists and are more likely to engage and persist in circumstances, and to spend more time on relevant tasks with better outcomes. However, considering the wide range of demands and activities in the setting of higher education, it is crucial that any attempt to intervene and improve accomplishment begin with understanding the students' individual needs in the learning environment.

## Supplementary Materials

For this article, the following Supplementary Materials are available:

- Study datasets. (Sepiadou, 2023)
- Study materials & measures. (Sepiadou, 2023)
- Study code. (Sepiadou, 2023)

## Data Availability

For this article data is available at Sepiadou (2023).
